# Responses of dry matter accumulation and partitioning to drought and subsequent rewatering at different growth stages of maize in Northeast China

**DOI:** 10.3389/fpls.2023.1110727

**Published:** 2023-03-20

**Authors:** Fu Cai, Na Mi, Huiqing Ming, Yushu Zhang, Hui Zhang, Shujie Zhang, Xianli Zhao, Bingbing Zhang

**Affiliations:** ^1^ Institute of Atmospheric Environment, China Meteorological Administration, Shenyang, China; ^2^ Key Laboratory of Agrometeorological Disasters, Liaoning, Shenyang, China; ^3^ Liaoning Province Meteorological Service Center, Shenyang, China; ^4^ Jinzhou Ecology and Agriculture Meteorological Center, Jinzhou, China

**Keywords:** maize, dry matter partitioning, drought response, total root biomass, root-shoot ratio

## Abstract

**Introduction:**

Dry matter accumulation (DMA) and dry matter partitioning (DMP) are important physiological processes determining crop yield formation. Deep understanding of the DMA and DMP processes and their responses to drought are limited by difficulty in acquiring total root biomass.

**Methods:**

Pot experiments with treatments quitting and ceasing ear growth (QC) and controlling soil water (WC) during vegetative (VP) and reproductive (RP) growth stages of maize (Zea mays) were conducted in Jinzhou in 2019 and 2020 to investigate the effects of drought and rewatering on DMW and DMP of different organs.

**Results:**

The response of DMW of reproductive organ to drought was more sensitive than those of vegetative organs, and was maintained after rehydration. Drought during VP (VPWC) reduced more sharply DMW of stalk than of leaves, and that during RP (RPWC) decreased more substantially leaves DMW. The effect of drought on DMPR was inconsistent with that on DMW for each organ. The DMP patterns of maize in different growth stages have adaptability to some level of water stress, and their responses increased with drought severity. Drought increased significantly DMP rates (DMPRs) of vegetative organs and reduced the ear DMPR and harvest index (HI), attributing to the suppressed photosynthates partitioning into ear and dry matter redistribution (DMRD) of vegetative organs, especially for stalk DMRD decreasing 26%. The persistence of drought impact was related to its occurrence stage and degree as well as the duration during rewatering to maturity. The aftereffect of drought during different growth periods on DMP were various, and that of VPWC enlarged and drastically induced the reduction of HI, also was larger than that of RPWC which demonstrated obvious alleviation in the previous responses of DMP and HI. Root-shoot ratio (RSR) increased under VPWC and RPWC and subsequent rehydration.

**Discussion:**

The DMWs of stalk, roots and leaves were affected by VPWC in order from large to small, and were close to or larger than the controls after rehydration, indicating the compensation effect of rewatering after drought. The DMPRs, RSR AND HI are the important parameters in agricultural production, and are often used as the constants, but in fact they vary with plant growth. In addition, the interannual differences in ear and stalk DMPRs in response to drought were probably caused by the difference in degree and occurrence stage of drought, further reflecting the variation in response of allometry growth among organs to the environment. Besides, the persistence of drought impact was related to the occurrence stage and degree of drought, which is also associated with the duration during rewatering to maturity. Notably, the effect of drought on DMW was inconsistent with that on DMPR for each organ meaning that the two variables should be discussed separately. The QC did not affect total DMW but increased RSR, changed and intensified the effect and aftereffect of RPWC on DMP, respectively, indicating that the DMP pattern and its response to drought occur change under the condition of QC.

## 1 Introduction

Dry matter accumulation (DMA) and dry matter partitioning (DMP) are important physiological processes determining crop yield formation (Kumar et al., 2006). In agro-ecosystems, crop yield is not only dependent on DMA, but also closely related to efficient allocation of dry matter to harvested organs (Zhang et al., 2019). The total amount of photosynthates stored in various organs at different growth stages of plants is determined by DMP and is affected by factors such as nutrition, temperature, radiation and soil moisture status (Steinfort et al., 2017; Lizaso et al., 2018). Generally, DMP refers to transport of accumulated photosynthates by leaves to different organs, which can be expressed as instantaneous values at a certain moment and cumulative values over a period of time (Poorter et al., 2012). According to the functional balance hypothesis, this allocation is characterized by preferential allocation of photosynthates to resource-constrained organs (Ma, 2017). In addition, when crops enter the reproductive period (RP), photosynthates mainly supply the growth of reproductive organs, while vegetative organs transfer a portion of dry matter to reproductive organs through dry matter redistribution (DMRD) to maintain a higher growth rate for the latter, with the redistribution rate of stalks being the largest, up to 35% of its dry weight (Weiner, 2004; Cai et al., 2022). Often, DMP is studied in plants such as crops and fruit trees whose reproductive organs are dominant (Andrews et al., 2001; Marcelis and Heuvelink, 2007). The ratio of DMP of photosynthates to each part of plant is usually calculated by measuring the change of dry matter weight (DMW) of each organ during a period of time. In crop models, aboveground and belowground biomass are often separated and the proportion of aboveground biomass occupied by each organ is then determined (Hijmans et al., 1994). In addition, root-shoot ratio (RSR) and harvest index (HI) are important parameters to reflect the DMP pattern among plant organs (Borras and Vitantonio, 2018), playing an important role in crop yield estimation and model construction.

Drought is one of the most vital constraints to crop yield and is an important factor affecting DMP (Li et al., 2019). On the one hand, it reduces dry matter quality by inhibiting photosynthesis (Gao et al., 2015) and, on the other hand, affects root and leaf growth by shifting DMP pattern (Berendse and Möller, 2009), thus affecting physiological processes such as nutrient absorption and photosynthesis (Djaman et al., 2013). Additionally, DMRD is inhibited by the ripening effect of drought (Turc and Tardieu, 2018). Overall, combination of the above effects leads to a decline in production. Studies on DMP have concentrated on the effects of such factors as planting density (Liu et al., 2011), sowing date (Dou et al., 2017), cultivation type (Wang et al., 2015; Cai et al., 2021) and soil fertilizer (Dai et al., 2008; Wei et al., 2017). There have also been some studies on the effects of drought on DMP (Jiang et al., 2018; Mi et al., 2018; Tan et al., 2019), but these have not generally considered roots, especially total root biomass, thus restricting in-depth understanding of relevant mechanisms.

Moreover, the response strategies of existing mainstream crop models to DMP under drought conditions all have defects of varying degrees, making it difficult to accurately simulate drought stress. In the AquaCrop model, allocation of photosynthates to different organs is not considered (Toumi et al., 2016). For the WOFOST model, the distribution of photosynthates to roots, stems and leaves is set to a fixed value only related to the development stage, and water stress would increase the proportion of roots, without considering DMRD (Hijmans et al., 1994). In the DSSAT model, the increase of DMW of leaves and stalks is proportional and not affected by environmental stress, and the proportion of DMRD at maturity is a fixed empirical parameter, without considering the effect of environmental stress (Lizaso et al., 2011). In the EPICphase model, water stress is considered, but DMP is empirically expressed and lacks a mechanism (Cavero et al., 2000). Therefore, deep investigation of the drought response mechanism of DMP and improving its parameter scheme are crucial to improve the ability of crop models to reproduce the drought process (Anothai et al., 2013).

Maize (*Zea mays*) is one of the three major food crops in the world, has the largest planting area and yield in recent years, and plays an important role in guaranteeing world food security and economic development (FAO, 2019). Northeast China is the main production area of spring maize in China, has the second largest maize belt in the world and plays a crucial role in grain production (Cheng et al., 2016). Maize is sensitive to its major growth constraint, drought, during the whole growth period (Mi et al., 2017; Huang et al., 2019; Zhou et al., 2020). Considering the limitation in understanding the response of crop DMP to drought duo to minor field experiment especially for scarce root measurement and the importance of maize in crop, we carried out 2-year pot experiment for maize suffering drought in order to integrally obtain DMWs of all organs and figure out how to be partitioned for maize photosynthate under drought and when the growth of maize ear is limited, which will offer abundant information about maize DMP pattern and further make up the shortage of the existing studies. Specifically, the objective of this study is to reveal (1) the variation characteristics of DMA, DMP and DMRD of different organs of maize at various growth stages under normal water supply; (2) their responses to drought and subsequent rehydration; and (3) the responses to QC treatment (quitting and ceasing ear growth during the RP), QC combined with drought, and subsequent rewatering. This study will enhance understanding of the disaster-causing process for maize under drought conditions, and promote improved parameter schemes in crop models, providing a scientific basis for accurately assessing the impact of drought and reasonably guiding disaster prevention and reduction for maize production.

## 2 Data and methods

### 2.1 Site description

The water control experiment for maize in present study was conducted at the Jinzhou Agricultural Meteorological Experimental Station in Liaoning Province, which has a temperate monsoon continental climate with average temperature and precipitation during 1981–2010 of 9.9°C and 568 mm, respectively. The study area has a typical brown soil with a pH 6.3 and nutritional composition including soil organic matter content of 15.24 g·kg^−1^, nitrogen of 1.04 g·kg^−1^, phosphorus of 0.50 g·kg^−1^ and potassium of 22.62 g·kg^−1^. The average field capacity, the wilting point and bulk density for the top 50 cm soil layer are 22.64%, 5.64% and 1.426 g·cm^−3^, respectively.

### 2.2 Experimental design

The experiment pots with sealed bases were made of PVC pipe with diameter 40 cm and height 100 cm, and were closely fixed row by row on the aboveground with a homemade metal fence, which formed a row and column interval of 40 cm among the plant. In the autumn before the experiment year, surface soil (0–20 cm) was evenly mixed, weighed and loaded into the pots. The soil water content was measured, and dry soil weight in pots was calculated. The maize variety was ‘Xianyu 335’. Three seeds were manually planted into the pots at the soil depth of 5 cm and a strong plant was remained when the corn had its fifth leaf. This research consisted of different experiments reflecting respectively the real-time and prolonged effects of drought. The experimental treatments and their abbreviations are shown in **Table 1**.

**Table 1:** Abbreviations used to denote each parameter and treatment.

Abbreviation	Description
DM	Dry matter
DMA	Dry matter accumulation
DMP	Dry matter partitioning
DMW	Dry matter weight
RSR	Root-shoot ratio
HI	Harvest index
DMPR	Dry matter partitioning rate
DMRD	Dry matter redistribution
WC	Water control treatment
VP	Vegetative period of maize
RP	Reproductive period of maize
QC	The treatment of quitting and ceasing ear growth during RP
CK	The control treatment
VPWC,VPCK	WC during VP and its CK
RPWC, RPCK	WC during RP and its CK
QCWC, QCCK	WC based on QC and its CK
VPAWC, RPAWC	VPWC and RPWC irrigated until maturity
CKA	The CK for VPAWC and RPAWC
QCAWC, QCACK	QCWC irrigated until maturity, and its CK
VPA, RPA, QCA	The corresponding referent treatments of VPAWC, RPAWC and QCAWC

The real-time effect experiment of drought included VPWC, RPWC and QCWC, as well as their corresponding control treatments: VPCK, RPCK and QCCK. More specifically, each treatment had six replicate pots. Natural precipitation was allowed before jointing, and appropriate amounts of water were added when precipitation was insufficient to ensure normal growth of maize plants. At jointing stage, six samples were selected to determine soil moisture: the average relative moisture of the soil column was 39.1 ± 4.0% and 49.4 ± 3.8% in 2019 and 2020, respectively. Then, water was replenished according to the difference between the measured and the optimum water content, i.e. relative soil moisture of 75%. After entering jointing stage, a large mobile waterproof shelter was used to prevent natural precipitation reaching the ground (Mi et al., 2018). According to the growth stage, weather and soil water conditions, the control treatment was irrigated with appropriate water to ensure the normal growth of maize. Treatments VPWC and RPWC reduced water supply from jointing to silking stage and from tasseling to milk ripening stage, respectively, to build drought episodes. Based on RPWC treatment, QCWC was conducted by wrapping female panicles in plastic bags to limit pollination and then to inhibit grain growth, in order to analyze the response of DMP in different organs under inhibited ear growth. Specifically, the DMWs of different organs of maize for each treatment were measured at the end of VPWC, RPWC and QCWC.

The prolonged effects of drought were reflected with comparisons of DMWs and DMPs of different organs after rewatering between drought and control treatments, and the experiment was designed as follows. After the end of drought process, some treatments for VPWC, RPWC and QCWC adopted the same irrigation measures as the control treatment until maturity, and were defined as VPAWC, RPAWC, and QCAWC, respectively, with the corresponding control treatments of VPAWC and RPAWC named CKA, and that of QCAWC named QCACK. Similarly, each treatment had six replicate pots.

**Tables 2**, **3** show the dates of growth periods of maize and irrigation regimes for the different treatments. In 2019, for the VPWC, water supplementation was not conducted on June 27, and was half of the amount of CKA from June 27 to July 15, and was the same as that of CKA after July 20. The DMWs for VPWC were observed on July 16. For the RPWC, water supplementation was not conducted on July 20, and was half of that of CKA from July 22 to August 14, and was the same as that of CKA after August 22. The DMWs for RPWC were observed on 15 August and harvesting was conducted on 15 September. In 2020, the water supplementation of VPWC was one-third of CKA for July 9–15, half of CKA on July 20 and consistent with CKA from July 24. The DMWs for VPWC were observed on July 21. For the RPWC, water supplementation was half of CKA from July 24 to August 11, and consistent with CKA after August 20. The DMWs for RPWC were observed on August 20 and harvesting was conducted on September 18.

**Table 2:** Dates (month/day) of maize growth periods and sampling in 2019 and 2020.

Growth/Sampling period	2019			2020		
	CKA	VPWC	RPWC	CKA	VPWC	RPWC
Sowing	4/30	4/30	4/30	5/10	5/10	5/10
Emergence	5/6	5/6	5/6	5/17	5/17	5/17
Jointing	6/15	6/15	6/15	6/20	6/20	6/20
Tasseling	7/10	7/12	7/10	7/16	7/16	7/16
Silking	7/15	7/17	7/15	7/21	7/27	7/21
Milk	8/6	8/6	8/6	8/18	8/18	8/18
Maturity	9/15	9/15	9/15	9/18	9/18	9/18
Sampling for VPWC	7/16			7/21		
Sampling for RPWC	8/15			8/20		

**Table 3:** Irrigation regimes for the different experimental treatments in 2019 and 2020.

Water supply amount (mm)	2019				2020			
	Dates (m/d)	CKA	VPWC	RPWC	Dates(m/d)	CKA	VPWC	RPWC
Precipitation	5/3-6/21	285.2	285.2	285.2	5/10-6/13	185.9	185.9	185.9
Irrigation	6/26	56.6	56.6	56.6	6/22	56.6	56.6	56.6
	6/27	28.3	0	28.3	6/25	5.7	5.7	5.7
	7/2	56.6	28.3	56.6	6/26	5.4	5.4	5.4
	7/5	56.6	28.3	56.6	6/29	85	85	85
	7/10	84.9	42.5	84.9	7/9	85	28.3	85
	7/15	56.6	28.3	56.6	7/15	85	28.3	85
	7/20	56.6	56.6	0	7/20	56.6	28.3	56.6
	7/22	56.6	56.6	28.3	7/24	56.6	56.6	28.3
	7/25	56.6	56.6	28.3	7/30	113.2	113.2	56.6
	7/29	28.3	28.3	28.3	8/3	113.2	113.2	56.6
	7/30	84.9	84.9	28.3	8/8	113.2	113.2	56.6
	8/1	56.6	56.6	28.3	8/11	113.2	113.2	56.6
	8/7	28.3	28.3	28.3	8/20	56.6	56.6	56.6
	8/14	56.6	56.6	28.3				
	8/22	56.6	56.6	56.6				
Total amount		1105.9	950.3	879.5		1131.2	989.5	876.5

### 2.3 Methods

#### 2.3.1 Soil water content measurement

Soil water content was calculated as follows:


(1) $$\theta_{rm}=\frac{\sum_{i}^{n}(\frac{\theta_{i}}{\theta_{f}})}{n}$$

where *θ_rm_* is soil relative moisture; *θ_i_* and *θ_f_* are soil weight water content and field capacity, respectively; and n is the number of replicates (i.e. n = 6).

**Figure 1:**
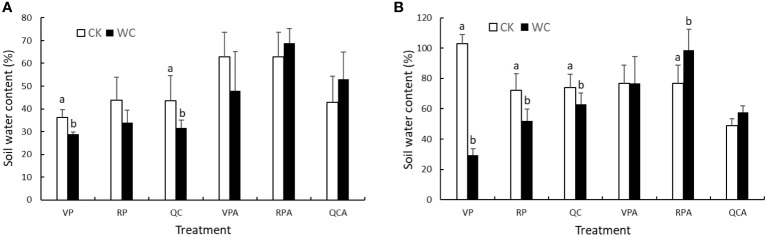
Soil moisture of WC and CK for different treatments in 2019 **(A)** and 2020 **(B)**.

Soil moisture status in pots at the end of different treatments in each year is shown in **Figure 1**. Soil moisture of the CK treatments was higher than those of the WC treatments after drought in 2019, but failed to reach the appropriate level, i.e. soil water content of 60%, due to deficient water supply. After rehydration, soil moisture of VPAWC, QCACK and QCAWC still did not reach 60%. Conversely, soil moisture of the CK treatment in 2020 reached the appropriate level after drought and rehydration, while soil moisture for QCAWC and QCACK was below 60% likely due to experimental errors. It is worth noting that soil moisture of RPAWC and QCAWC was higher than for the corresponding CK treatments in both years. The reason was that the physiological functions of plants were disrupted due to RP drought, and the plants withered after rewatering, which decreased the water consumption of transpiration. In addition, the measured soil samples were in the outer layer of the soil column, and were drier than those in the inside of the soil column, resulting in a lower value relative to the real condition.

#### 2.3.2 Sampling and measurement

The height, stalk diameter and leaf area of maize plants were measured at the end of every treatment. The stalk diameter was presented with the maximum width of the second stem node from ground surface. Maximum length and width were measured for each leaf of the maize plant, and computation formulas of total leaf area per plant (LA) are as follows:


(2) $$\text{LA}=\sum_{i=1}^{\text{n}}(L_{i}\times W_{i}\times 0.75)$$

Where *i* is the number of leaves on the plant, *L_i_* is the maximum leaf length, *W_i_* is the maximum leaf width, and 0.75 is a factor used conventionally.

Plants from each treatment were cut at the ground level and separated into the stalk, leaves, bracts and ear. It should be noted that the ear was divided into kernel and cob to measure in 2019, but not in 2020. Roots were obtained with washing method. Specifically, the experiment pot was cut open to gain an intact soil pillar, and then the soil pillar was splitted into segments in an interval of 10 cm, and was soaked in water for a period of time. At last, all roots were gained with washing. All the samples were oven-dried at 105°C for 30 min, and weighed after drying at 70°C to constant weight (Mi et al., 2018).

#### 2.3.3 Calculations of DMPR, RSR and HI

In order to reflect the relationship of DMP among different organs and their responses to drought, the variation characteristics of DMPRs, RSRs and HIs were studied.

DMPR is expressed as follows:


(3) $${\text{DMPR}}_{\text{i}}{\text{= DM}}_{\text{i}}{\text{/DM}}_{\text{t}}$$

$${\text{DM}}_{\text{t}}{\text{= DM}}_{\text{r}}{\text{+ DM}}_{\text{s}}{\text{+ DM}}_{\text{l}}{\text{+ DM}}_{\text{e}}{\text{+ DM}}_{\text{b}},$$

where DM_i_ is the DMW of organ i; DM_r_, DM_s_, DM_l_, DM_e_ and DM_b_ are the DMWs of roots, stalks, leaves, ears and bracts, respectively and DMPR_i_ is the DMPR of organ i.

RSR is expressed as follows:


(4) $${\text{RSR = DM}}_{\text{r}}{\text{/DM}}_{\text{ab}},$$

where DM_ab_ is aboveground DMW, i.e. the sum of DMWs of stalks, leaves, ears and bracts.

HI is expressed as follows:


(5) $${\text{HI = DM}}_{\text{g}}{\text{/DM}}_{\text{ab}}.$$

DM_g_ is the DMW of grain i.e. the DM_e_ substracts the DMW of maize cob. The ratio of DM_g_ to DM_e_ for RPWC, RPCK, VPAWC, RPAWC and CKA in 2019 were 0.82, 0.85, 0.82, 0.84 and 0.87, and were used to calculate corresponding DM_g_ in 2020.

### 2.4 Data statistics

The observation data each year were statistically analyzed using SPSS 20.0 software (SPSS Inc., Chicago, IL, USA, URL:(https://www.ibm.com/cn-zh/spss?lnk=flatitem)) separately. The differences among experimental treatments were calculated using Duncan’s multiple comparison test and a one-way ANOVA at the 0.05 significance levels.

## 3 Results

### 3.1 Drought response of maize morphological characteristics

**Figure 2:**
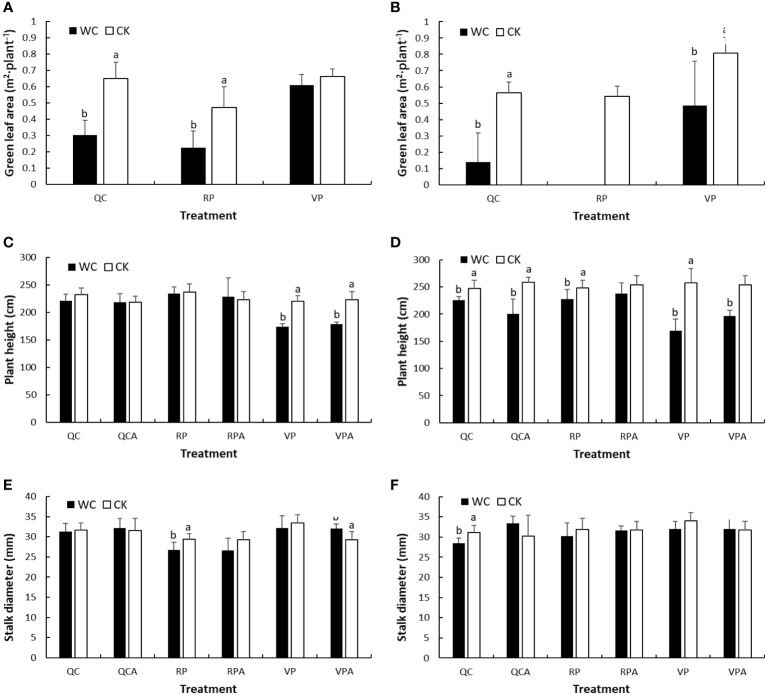
Green leaf area **(A, B)**, plant height **(C, D)** and stalk diameter **(E, F)** for WC and CK of different treatments in 2019 and 2020.

**Figure 2** shows the green leaf area per plant, plant height and stalk diameter for WC and CK after different treatments. Different letters represent significance level (P < 0.05) of differences of the variables between WC and CK treatments; no letter indicates insignificant difference; also applies in the other figures. The green leaf area of RPWC in 2020 was 0 because the leaves were all dry and not green. In 2019 and 2020, the leaf areas of most WC treatments were significantly smaller than those of the controls except that of VPWC in 2019.

For plant height, there were no significant differences between WC and CK for RP, RPA, QC and QCA treatments in 2019, while those for VP and VPA were significant. In 2020, RPWC resulted in a significant small reduction in plant height, and rewatering narrowed the difference at later growth stages. The QCWC significantly decreased plant height, and its effect increased in later growth stages. Both VPWC and VPAWC induced significant and sharp decreases in plant height.

In 2019, stalk diameter was insignificantly reduced after VPWC, and significantly increased after rewatering. The RPWC significantly reduced stalk diameter, but the reduction was insignificant after rewatering. There were no significant variations in stalk diameter after QCWC and following rehydration. In 2020, stalk diameter was insignificantly affected by VPWC and RPWC, and reduced significantly after QCWC, but insignificantly increased for QCAWC.

### 3.2 Responses of maize DMWs to drought

#### 3.2.1 Responses of belowground, aboveground and total DMWs to drought

**Figure 3:**
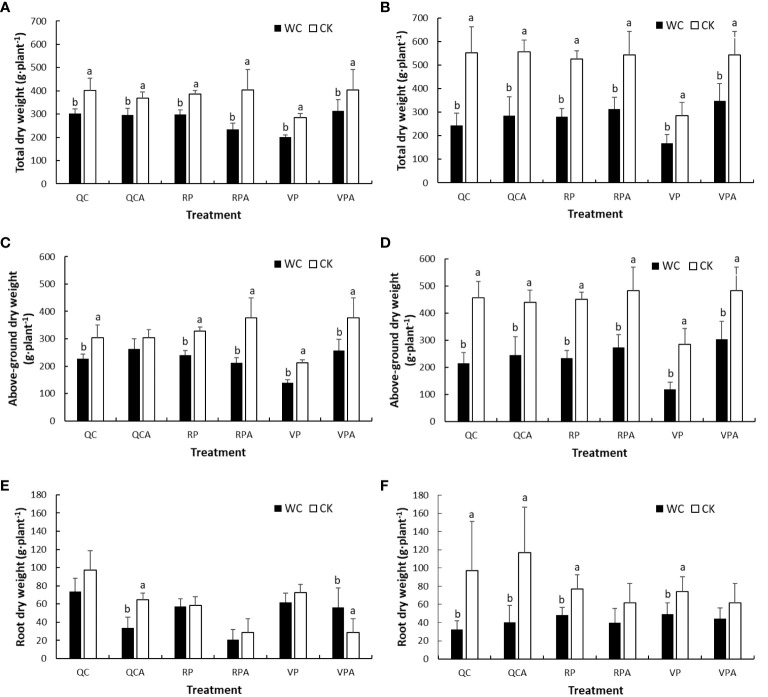
Comparisons of total **(A, B)**, aboveground **(C, D)** and root **(E, F)** DMWs of maize between WC and CK for different treatments in 2019 and 2020.

The aboveground and total DMWs of maize for different drought treatments in 2019 and 2020 were nearly all significantly lower than the corresponding control values (**Figure 3**). They increased significantly from the end of VPWC to maturity, and the differences in DMWs of all treatments between WC and CK in 2020 were greater than in 2019. The roots DMWs of CK showed a decreasing trend from the end of VPWC and RPWC to maturity. The roots DMWs of VPWC and VPAWC were slightly smaller and significantly greater than the controls in 2019, respectively, and correspondingly were significantly and insignificantly smaller in 2020. The roots DMWs of RPWC and RPAWC were unaffected and insignificantly smaller than the control in 2019, respectively, and correspondingly significantly smaller and not significantly different in 2020. Under normal water supply, there was no significant difference in total DMW between QC and non-QC treatments in 2019 and 2020, belowground and aboveground DMWs of QC treatment were higher than and similar to those of non-QC treatment, respectively. The belowground DMWs of QCWC and QCAWC were slightly and significantly smaller than the controls in 2019, respectively, but both significantly smaller in 2020.

#### 3.2.2 Responses of DMWs of aboveground organs to drought

**Figure 4:**
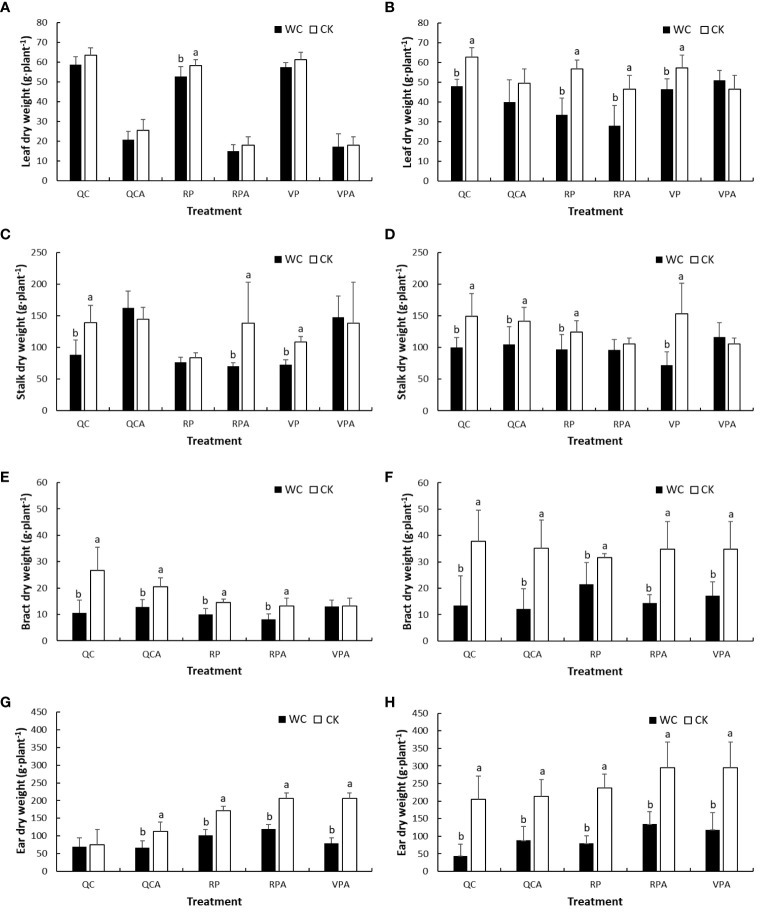
Comparisons of DMWs of aboveground organs of maize i.e. leaf **(A, B)**, stalk **(C, D)**, bract **(E, F)** and ear **(G, H)** between WC and CK for different treatments in 2019 and 2020.

The leaf DMWs of different control treatments in the 2 years were lower at maturity than after drought (**Figure 4**), indicating that they decreased with development progress of maize in the natural state, and the decline was significantly greater in 2019 than 2020. From the perspective of drought effect, in 2019, the leaves DMW of RPWC decreased significantly, while those of the other WCs were slightly and insignificantly smaller than the control. In 2020, the leaves DMWs of most WCs were significantly smaller and that of VPAWC was insignificantly larger than the control. Stalk DMW, in 2019, was significantly lower for VPWC and QCWC and slightly larger for VPAWC and QCAWC under drought than the control values. There was little difference in stalk DMW between RPWC and RPCK, but the stalk DMW of RPAWC decreased significantly. In 2020, the stalk DMWs of most WCs except for RPAWC and VPAWC decreased significantly relative to the control. For bracts, most WCs except for VPAWC in 2019 significantly reduced DMWs in both years. The controls in 2019 were all smaller than those in 2020, and the decrease ranges of bracts DMWs for WCs in 2020 were significantly greater than those in 2019. The maize ear DMW for VP was not analyzed because the ear was not formed after VP. Specifically, the maize ears of most WCs except for QCWC in 2019 were significantly lower than the controls in both years, and with obviously greater reductions in 2020 than 2019. Notably, under normal conditions, the ear DMW of QCCK was lower than that of RPCK, demonstrating that QC inhibited the growth of ears.

### 3.3 Responses of RSR and HI to drought

**Figure 5:**
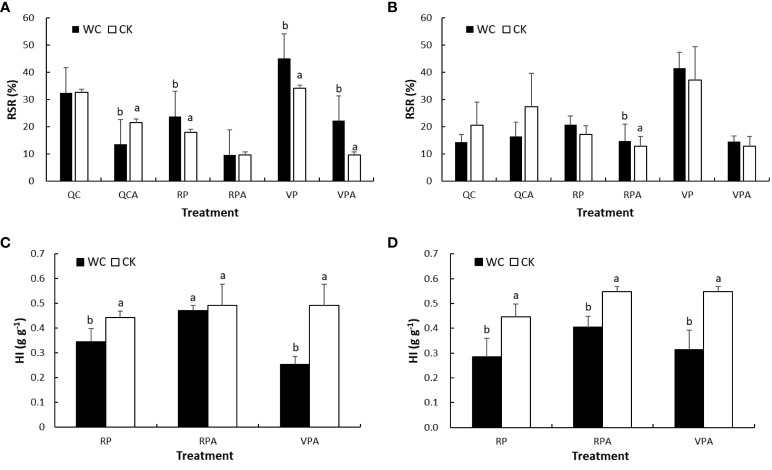
Comparisons of RSR **(A, B)** and HI **(C, D)** between WC and CK for different treatments in 2019 and 2020.

The RSRs of VPWC and VPAWC were significantly 32% and 132% higher than those of CKs in 2019, respectively, and correspondingly in 2020 were insignificantly higher by 12% and 13% (**Figure 5**). The RSR of RPWC increased significantly by 33% and insignificantly by 22%, while those of RPAWC varied slightly and significantly increased by 14% compared with the control in 2019 and 2020, respectively. However, the RSRs of QCCK were 82% and 20% larger than those of RPCK in 2019 and 2020, respectively. The RSRs of QCACK were 125% and 112% larger than those of CKA in 2019 and 2020, respectively. Whereas, the RSRs of QCWC and QCAWC were very similar and significantly 37% smaller compared with the controls in 2019, respectively, and correspondingly insignificantly 31% and 40% less in 2020.

The DMWs of grain for different treatments in 2020 were calculated with the ratio of grain to ear DMWs from the experiment in 2019 based on small variability for these ratios, and further HIs in 2020 were obtained. Comparing HIs between WCs and CKs showed that those for VPAWC and RPWC were significantly lower than the controls in 2019 and 2020. The HIs of RPAWC in 2019 and 2020 were insignificantly and significantly lower than the controls, respectively. The reduction of HIs in descending order was VPAWC, RPWC and RPAWC for both years.

### 3.4 Effect of drought on maize DMP

**Figure 6:**
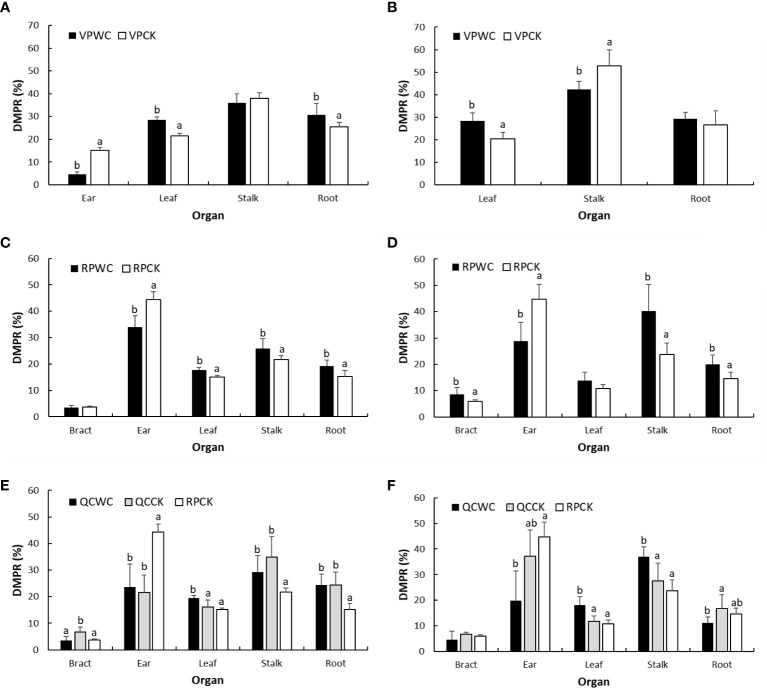
Comparisons of DMPRs of maize organs of different treatments for VP **(A, B)**, RP **(C, D)**, and QC **(E, F)** between WCs and CKs in 2019 and 2020.

At the end of VPWC, maize was at silking stage in 2019 and 2020. The ear, leaves, stalk and roots DMPRs of VPCK in 2019 were 15%, 21%, 38% and 25%, respectively, and the leaves, stalk and roots DMPRs of VPCK in 2020 were 21%, 52% and 27%, respectively, when the ear and stalk DMWs were considered together as stalk DMW (**Figure 6**). After VPWC, the roots and leaves DMPRs significantly increased by 21% and 33%, respectively, the ear DMPR decreased significantly by 70%, and the change of stalk DMPR was not obvious in 2019; the leaves and stalk DMPRs increased and decreased significantly by 38% and 20%, respectively, and the root DMPR increased insignificantly by 10% in 2020. We speculate that the decrease of stalk DMPR in 2020 was mainly due to the reduction of ear DMW.

At the end of RPWC, the maize was at 16 and 2 days after milk ripening in 2019 and 2020, respectively. The DMPR of each maize organ for RPCK was 4% in bracts, 44% in ear, 15% in leaves, 22% in stalk and 15% in roots in 2019, and correspondingly 6%, 45%, 11%, 24% and 14% in 2020. The DMPRs of most of maize organs were significantly changed by drought stress. Specifically, ear DMPR significantly decreased by 24% and 36% in 2019 and 2020, respectively, and leaves, stalk and roots DMPRs increased by 17%, 19% and 27% in 2019, respectively, and by 27%, 69% and 37% in 2020; the increase in leaves DMPR in 2020 was insignificant. In addition, bracts DMPR was unaltered in 2019 and increased significantly by 42% in 2020 relative to the control.

Comparing the DMPR between QCCK and RPCK treatments showed that QC significantly reduced ear DMPR in 2019, but significantly increased the bracts, stalk and roots DMPRs. In 2020, the reduction in ear DMPR of QCCK relative to RPCK was less than that in 2019, and the DMPRs of other organs increased slightly. The DMPR of ear increased slightly for QCWC compared with QCCK, that of stalk decreased insignificantly, that of roots did not change, that of bracts significantly decreased and that of leaves significantly increased in 2019. The DMPRs of bracts and ear decreased insignificantly, that of roots decreased significantly and those of leaves and stalk increased significantly in 2020.

### 3.5 Continuity of drought effect on maize DMP

**Figure 7:**
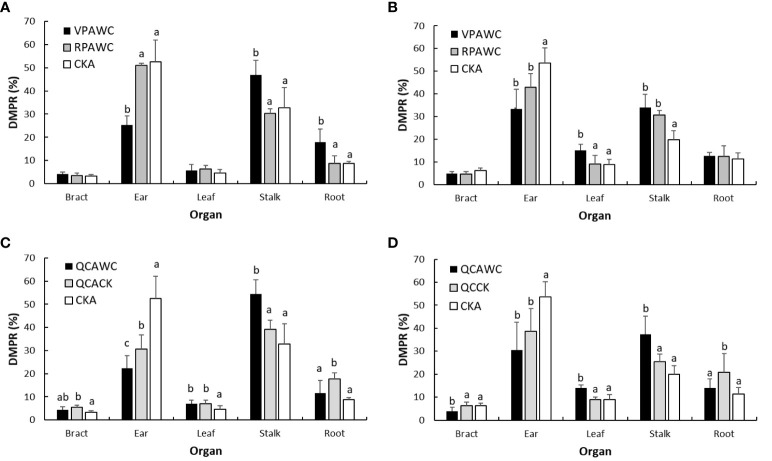
DMPRs of maize organs at the end of growth for WC **(A, B)** and QC **(C, D)** treatments in 2019 and 2020.

The effect of previous drought on following maize growth can be shown by comparing the DMPRs of each organ between WC and CK at maturity. For VPAWC, the DMPR of ear decreased significantly, of stalk and roots increased significantly, and of bracts and leaves did not change significantly relative to the control in 2019 (**Figure 7**). In 2020, the DMPR of ear decreased, those of leaves and stalk increased significantly and that of roots was almost unchanged relative to the controls. Compared with the effects of VPWC, the difference between VPAWC and CKA in leaves DMPR decreased, while those in the DMPRs of other organs increased obviously in 2019. In 2020, the differences in DMPRs for ear and stalk increased, and those of other organs were unchanged.

For RPAWC, there was no significant difference in the DMPR of maize organs between WC and CK in 2019, and the DMPRs of ear, stalk and other organs were significantly smaller, larger and unchanged relative to the controls in 2020, respectively. Compared with the effects of RPWC, in 2019, the differences in DMPRs of each organ between RPAWC and CKA were obviously reduced. In 2020, the differences in bracts and roots DMPRs obviously decreased, and were unchanged for ear, leaves and stalk DMPRs.

In terms of QCACK, compared with CKA, the DMPRs of bracts, leaves and roots increased significantly, that of stalk increased insignificantly and that of ear significantly decreased in 2019. The DMPRs of bracts and leaves did not change, of ear decreased significantly, of stalk increased insignificantly and of roots increased significantly in 2020. Compared with QCACK, the DMPRs of bracts and leaves for QCAWC had no significant change, of ear and roots significantly decreased and of stalk significantly increased in 2019. The DMPRs of bracts and roots significantly decreased, of ear decreased insignificantly and of leaves and stalks significantly increased in 2020.

Relative to difference between RPAWC and CKA, the differences in the DMPRs of ear, stalk and roots between QCAWC and QCACK increased in 2019. However, the differences in the DMPRs of bracts, leaves and roots increased in 2020. Compared with the effects of QCWC, the differences between QCAWC and QCACK in the DMPRs of ear and roots changed from being inconspicuous to significantly decreasing, that of stalk changed from decreasing to significantly increasing, and those of leaves and bracts changed from significantly increasing and decreasing, respectively, to no significant difference in 2019. The differences in the DMPRs of different organs in 2020 were invariable.

### 3.6 Drought response to DMRD process

#### 3.6.1 Potential DMRD capacity of maize vegetative organs

Because there was no one-to-one correspondence between the sample plants at the end of drought and growth, the subtraction of the mean value of six samples at the two times was used to express the increment of DMW without the sample variance. It is well-known that vegetative organs including stalk, leaves and roots of maize reach their maxima at tasseling and silking stage, after which some dry matter is transferred to ear through a redistribution process. Notably, total DMW of maize plant of CK was sharply smaller in 2019 than 2020, indicating that the maize of CK in 2019 was also subjected to water stress in part of the period. As a result, the analysis was only conducted considering the situation in 2020. Under normal water supply, the decreased DMW of each vegetative organ was approximately equal to the DMRD amount during tasseling to maturity (from July 21 to September 18). The DMRD rates of different vegetative organs are shown in **Table 4**. About 35%, 41%, 24% and 27% of DMWs in total vegetative organs, stalk, leaves and roots were redistributed to ear, respectively, accounting for 31%, 21%, 4% and 6% of the increment of ear DMW. The contributions of stalk, leaves and roots were 65%, 13% and 22%, respectively.

**Table 4:** Potential percentage and contribution of maize vegetative organs to DMRD.

Parameters	Stalk	Leaves	Roots	Total
Percentage of organs DMW for redistribution (%)	41	24	27	35
Proportion of organ redistribution accounting for the increment of ears DMW (%)	21	4	6	31
Contribution of organ redistribution to the increment of ears DMW (%)	65	13	22	100

‘Total’ stands for total amount of DMRD of vegetative organs i.e. stalk, leaves and roots.

#### 3.6.2 Drought response of DMRD capacity of maize vegetative organs

The DMRDs for RPWC and RPCK from August 20 to September 18 are shown in **Table 5**. Under RPCK, during this period, 27% of stalk, 20% of leaves, 32% of roots and 27% of total vegetative organ DMWs were redistributed into ear. The ratios of DMRD from stalk, leaves and roots to increment of ear DMW were 34%, 11% and 23% accounting for 51%, 16% and 33% of DMRD of total vegetative organs, respectively. Under RPWC, 9% of DMW of total vegetative organs consisting of 1% of stalk, 20% of leaves and 22% of roots DMWs, accounting for 32%, 2%, 12% and 18% of increment of ears DMW, respectively, was redistributed into ear. The contributions of stalk, leaves and roots were about 7%, 36% and 57%, respectively. The proportions of stalk and roots DMRDs decreased to varying degrees due to drought, and the decrease for stalk was the largest, reflecting that drought seriously affected dry matter transfer from stalk to ear. The VPWC severely inhibited the redistribution from vegetative organs from tasseling to maturity. Specifically, the drought-induced limitation in DMA led to increases of stalk and leaves DMWs due to a compensatory effect during this period, and then their DMWs at maturity were significantly higher than those at tasseling stage and so their DMRDs could not be quantitatively determined.

**Table 5:** Proportion and contribution of DMRD in maize organs.

Treatment	Parameters	Stalk	Leaves	Roots	Total
RPCK	Percentage of vegetative organs DMRD (%)	27	20	32	27
	Proportion of organ DMRD accounting for the increment of ears DMW (%)	34	11	23	68
	Contribution of organ DMRD to the increment of ears DMW (%)	51	16	33	100
RPWC	Percentage of vegetative organs DMRD (%)	1	20	22	9
	Proportion of organ DMRD accounting for the increment of ears DMW (%)	2	12	18	32
	Contribution of organ DMRD to the increment of ears DMW (%)	7	36	57	100

## 4 Discussion

As an important concept in the fields of ecology and agriculture, DMP varies with changes in the environmental situation (Borras and Vitantonio, 2018). Although DMPR is the key parameter in crop models, existing models fail to determine the relationship between DMPR and environmental factors, which directly affects the simulation accuracy of biomass for different plant organs (Cai et al., 2022). In this paper, the biomasses of aboveground and belowground organs of maize were collected in a pot experiment under drought stress, and the drought response of DMP of each organ and compensation effect of following rehydration were investigated.

### 4.1 Responses of main morphological characteristics of maize to drought and rewatering

The change of morphological characteristics is the most direct manifestation of maize affected by drought (Welcker et al., 2011), which lowers the physiological function of leaves and further influences crop growth and yield (Chaves et al., 2009; Lobell et al., 2014). Droughts during different periods of maize growth affected green leaf area. As the plant growth center was transferred to the reproductive organ and the leaves gradually senesced, RPWC accelerated senescence of leaves and decreased green leaf area sharply compared to VPWC, related to decreasing photosynthesis capacity with the progress of growth (Song et al., 2018). In addition, drought affected two aspects of stalk growth: plant height and stalk diameter. Specifically, plant height was mainly influenced by drought in VP (Song et al., 2018). After rewatering, stalk growth was compensated by increasing diameter, reflecting that the plant adapted to environmental stress through varying its morphology (Lamers et al., 2020). RPWC slightly affected plant height, but significantly decreased stalk diameter (**Figure 2**), resulting in a decrease in stalk DMW (**Figure 4**).

### 4.2 Responses of DMWs of maize organs to drought

Drought induces the change in the DMP pattern, which is conducive to drought resistance (Shipley and Meziane, 2002). Studies on dry matter have mostly focused on plant aboveground parts, with very limited attention to total DMW due to the difficulty in acquiring whole roots (Mccormack et al., 2015; Julia et al., 2016; Komainda et al., 2016). In this study, the drought regime was artificially manufactured based on an experiment conducted throughout the whole growth process of maize using large experimental pots to allow whole roots sampling. The aboveground and total DMWs decreased significantly due to drought in different periods, and the DMWs of the various organs decreased to different degrees, reflecting differences in response to drought. The DMWs of stalk, roots and leaves were affected by VPWC in order from large to small, and were close to or larger than the controls after rehydration, indicating the compensation effect of rewatering after drought (Zhen and Wang, 2018). The effects of RPWC in descending order were leaves, roots and stalk, and the compensative growth of each organ to rehydration was not obvious, which was related to the short duration from rehydration to maturity and the gradual senescence. Notably, there was no significant difference between QC and non-QC treatments in total DMW, indicating that QC had no effect on total maize biomass. The DMWs of roots, stalk, leaves and bracts under QCCK were significantly higher than those under non-QC treatment, especially for roots and stalk, indicating that inhibition of ear growth could increase DMWs of vegetative organs. For QCWC, the DMW of each organ was significantly lower than the control, with rehydration playing a limited role in decreasing drought influence.

### 4.3 Drought responses of RSR and HI at different growth stages

The DMPs of different plant organs vary with growth stage and environment (Yin et al., 2016; Hu et al., 2022). In this study, VPWC and RPWC both increased RSR, an important indicator of crop yield (Liu et al., 2017), showing that drought promoted DMP to roots, consistent with the functional balance theory (Shipley and Meziane, 2002). The RSRs of QCCK and QCACK were larger than of RPCK and CKA, respectively (**Figure 5**), meaning that the dry matter originally allocated to ear would be allocated to other organs, especially roots, and the response of DMP to drought would be different from non-QC treatment, that is, the RSR decreased instead of increased. The HI, also known as reproductive effort in ecology, is an important parameter to measure crop productivity in agricultural production, and is often used as a constant to estimate yield by multiplication with aboveground DMW (Maddonni, 2012), but in fact it varies with plant growth (Bonelli et al., 2016). The RPWC reduced HI in different degrees, and rehydration had a recovery effect on HI. The HI of VPAWC was smaller than that of RPAWC, meaning that VPWC had a greater aftereffect on HI than RPWC.

### 4.4 Drought responses of DMP in maize at different growth stages

Further study showed that mild water stress had a limited effect on the DMP pattern of maize during VP, reflecting that DMP of maize has adaptability to some level of water stress. When VPWC was aggravated, the DMPRs of roots and leaves increased, DMP of ear was inhibited (Yu et al., 2009). For RPCK, there was also good interannual consistency in the DMP pattern, but drought reduced the DMPR of ear, and increased the DMPRs of other organs because, on one hand, drought suppressed photosynthates partitioning into ear and, on the other hand, DMRD from vegetative organ was inhibited (Dang et al., 2014). In addition, the interannual differences in ear and stalk DMPRs in response to drought were probably caused by the difference in degree and occurrence stage of drought (Wang et al., 2016), further reflecting the variation in response of allometry growth among organs to the environment (Zhang et al., 2019). The DMP for QCCK showed that the dry matter originally allocated into ear will be allocated to stalk and roots. QC also changed the effect of drought during RP on DMP pattern. Specifically, QCWC significantly increased leaf DMP, and affected the DMPs of other organs differently due to the difference of the extent of ear inhibition between years.

### 4.5 Continuity of drought effect on DMP in maize at different growth stages

Deep understanding of the aftereffect of previous drought on maize growth has an important role in drought impact prediction (Luo et al., 2016). The previous drought during different growth periods had various significant aftereffects on DMP of maize after rewatering. The aftereffect of VPWC had temporal difference among maize organs and varied with the growth process in different years (Mi et al., 2017). However, rehydration alleviated the response of DMP of each organ to RPWC. Thus, the persistence of drought impact was related to the occurrence stage (Cai et al., 2020) and degree of drought (Alam et al., 2014; Cai et al., 2022), and was weaker in RP than in VP, which is also associated with the duration during rewatering to maturity. Regrettably, soil water content was not continuously measured and so the influence of drought degree could not be evaluated in this study. Furthermore, rehydration after QCWC caused remarkable reduction in DMPR of ear and roots and increased stalk DMPR. Besides, the QC intensified the aftereffect of drought in RP on the DMP. Notably, the effect of drought on DMW was inconsistent with that on DMPR for each organ (Shi et al., 2021), meaning that the two variables should be discussed separately.

### 4.6 Drought response of maize DMRD

The DMRD of vegetative organ is an important source of DMA of reproductive organ during late maize growth period (Weiner et al., 2009). Potential DMRD is the key parameter in the DMP process of crop models, and plays a crucial role in accurately estimating crop yield (Dang et al., 2014). Ma and Zhou (2016) set the redistribution potential of stalk, leaves and roots as 30%, 10% and 10%, respectively, while those in this study were relatively higher. The DMRD potential of stalk was significantly higher than those of leaves and roots, and the latter two were similar to each other. Under normal growth conditions, from tasseling to maturity, the contributions to ear DMA were in descending order of stalk, roots and leaves. Whereas, the DMRDs of stalk and roots decreased sharply and slightly under drought, respectively, probably due to plant senescence and leaf abscission, indicating that drought had an inhibitory effect on the DMRD of stalk and roots. Liu et al. (2006) found that the DMPRs of maize roots and leaves at maturity significantly decreased by more than 1 times relative to those in silking stage, which is consistent with results in this study.

## 5 Conclusions

In this study, the effects of drought on DMA and DMP of maize organs during VP and RP were studied based on an experiment using large capacity pots. The responses to drought and following rehydration for the DMAs, DMPRs, redistribution potential, RSRs and HIs of different organs were deeply analyzed with some conclusions obtained as follows.

The DMAs of maize organs declined under drought in different growth periods. The VPWC had larger effect on the DMW of stalk than of leaves, and rehydration resulted in compensatory growth of stalk, leaves and roots. The RPWC affected green leaf area and leaves DMW more significantly than did VPWC. The effects of VPWC and RPWC and following rehydrations on roots DMWs were very similar. Bracts and ear DMWs were sensitive to drought during different periods, and their reductions were greater than those of vegetative organs. The QCCK did not affect total DMW of maize plants, leading to more dry matter transfer to roots. Whereas, QCWC and QCAWC reduced significantly DMW of each organ relative to QCCK. The DMP pattern and RSR of maize organs in different growth stages maintained certain stability under normal water supply or mild drought, and varied and increased with aggravation of drought, respectively. The RSR of QC was larger than that of non-QC under normal conditions, and declined under drought, which is opposite to the effect of non-QC drought. Rewatering increased (decreased) the responses of RSR of QC (non-QC) to drought. The VPWC increased DMPRs of roots and leaves, and decreased ear DMPR and did not change stalk DMPR. After rewatering, HI was still dramatically smaller than the control, but the DMPR of stalk significantly increased. The RPWC reduced HI and ear DMPR and increased the DMPRs of vegetative organs. However, rehydration alleviated reductions of HI and the response of DMP of each organ to drought. The QC intensified the aftereffect of previous RPWC on the DMPRs. The potential of DMRD of stalk was larger than those of leaves and roots. The contribution of DMRD of vegetative organs to ear DMA during milk ripening to maturity in descending order were stalk, roots and leaves. Drought inhibited sharply and slightly the redistribution of stalk and roots, respectively.

## Data availability statement

The raw data supporting the conclusions of this article will be made available by the authors, without undue reservation.

## Author contributions

FC and YZ conceived and designed the experiments, and FC drafted the manuscript; HZ, BZ and XZ performed the experiments; NM, HM and SZ analyzed the data and prepared all figures. All authors contributed to the article and approved the submitted version.
